# How theory can help to understand the potential impact of food environment policies on socioeconomic inequalities in diet: an application of Bourdieu’s capital theory and the scarcity theory

**DOI:** 10.1093/eurpub/ckac052

**Published:** 2022-11-29

**Authors:** Sanne K Djojosoeparto, Carlijn B M Kamphuis, Janas M Harrington, Anne Lene Løvhaug, Gun Roos, Alexia D M Sawyer, Karien Stronks, Laura Terragni, Liv Elin Torheim, Stefanie Vandevijvere, Maartje P Poelman, Frank J van Lenthe

**Affiliations:** Department of Human Geography and Spatial Planning, Faculty of Geosciences, Utrecht University, Utrecht, The Netherlands; Department of Interdisciplinary Social Science, Faculty of Social and Behavioural Sciences, Utrecht University, Utrecht, The Netherlands; School of Public Health, University College Cork, Cork, Ireland; Department of Nursing and Health Promotion, OsloMet—Oslo Metropolitan University, Oslo, Norway; Consumption Research Norway (SIFO), OsloMet—Oslo Metropolitan University, Oslo, Norway; Department of Public and Occupational Health, Amsterdam UMC, University of Amsterdam, Amsterdam, The Netherlands; Department of Public and Occupational Health, Amsterdam UMC, University of Amsterdam, Amsterdam, The Netherlands; Department of Nursing and Health Promotion, OsloMet—Oslo Metropolitan University, Oslo, Norway; Department of Nursing and Health Promotion, OsloMet—Oslo Metropolitan University, Oslo, Norway; Department of Physical Health and Ageing, Norwegian Institute of Public Health, Oslo, Norway; Service of Lifestyle and Chronic Diseases, Sciensano, Brussels, Belgium; Chair Group Consumption and Healthy Lifestyles, Department of Social Sciences, Wageningen University & Research, Wageningen, The Netherlands; Department of Human Geography and Spatial Planning, Faculty of Geosciences, Utrecht University, Utrecht, The Netherlands; Department of Public Health, Erasmus University Medical Center Rotterdam, Rotterdam, The Netherlands

## Abstract

Government policies that promote healthy food environments are considered promising to reduce socioeconomic inequalities in diet. Empirical evidence of effects on these inequalities, however, is relatively scarce and, with a few exceptions, tends to be inconclusive. We use two contemporary theories that help to understand socioeconomic inequalities in health and health-related behaviours (Bourdieu’s capital theory and Mullainathan and Shafir’s scarcity theory) to reason how policies influencing food environments may differentially impact lower and higher socioeconomic groups. In essence, these theories enable us to understand how specific elements of broader daily living conditions (e.g. social practices that lead to habitus formation, material conditions that shape experiences of scarcity) may lead to a greater benefit of certain food environment policies for the healthfulness of diets of lower or higher socioeconomic groups. We conclude that the application of theories on the mechanisms underlying socioeconomic inequalities in health can help to guide future empirical studies in testing theory-based hypotheses on differential effects of policies, and thereby enhance the development of effective policies tackling socioeconomic inequalities in dietary intakes.

## Introduction

Socioeconomic inequalities in diet are observed across the world and in many European countries.^1^^,^^2^ Overall, lower socioeconomic groups report less healthy dietary intakes than higher socioeconomic groups (e.g. lower fruit and vegetable consumption, higher intake of energy-dense foods),^1^ which contributes to higher prevalence rates of obesity and diet-related chronic diseases^3^ among lower socioeconomic groups.^4^ Increasing patterns of inequalities in dietary intake over the past decades have coincided with large and detrimental changes in the food environment.^4^ Food environments are defined as the collective physical, economic, policy, sociocultural and commercial surroundings, opportunities and conditions that influence people’s food and beverage choices and nutritional status.^5^ An easy availability and prominent marketing of cheap, ultra-processed, energy-dense and nutrient-poor food products, currently characterize food environments of high-income countries and increasingly those of middle-come countries as well.^6^ As a result, diets have become less healthy in most regions of the world,^4^ and apparently more in lower than higher socioeconomic groups.^1^^,^^2^

Government policies that lead to healthier food environments are considered promising to reduce socioeconomic inequalities in diet.^7^ Such policies are specified in the Healthy Food-Environment Policy Index (Food-EPI), which was developed based on high level international recommendations and expert consultations.^7^ The Food-EPI specifies important policy domains and good practice indicators via which governments can improve the healthfulness of food environments, e.g. by regulating the availability, accessibility and quality of foods in shops, schools, workplaces; or by price reductions of healthy foods.^5^^,^^7^ The seven policy domains included in the Food-EPI are food composition, food labelling, food promotion, food prices, food provision, food in retail, and food trade and investment.^7^

Food-EPI related governmental policies are mainly universal by nature; whole populations, rather than specific parts of populations, are exposed to their implementation. Universal interventions that make changes to the structural environment (e.g. food marketing policies) are considered more likely to reduce health inequalities than individual-level interventions (e.g. health mass media campaigns).^8–10^ However, empirical evidence on the differential impact of food environment policies for lower and higher socioeconomic population groups is scarce.^10^^,^^11^ A recent umbrella review showed that most research on this has been done regarding food price policies, i.e. economic measures to incentivize healthy or disincentivize unhealthy food purchases.^11^ The review concluded that taxation of unhealthy foods and beverages, and food-related income support programmes for lower socioeconomic groups, may reduce socioeconomic inequalities in diets.^11^ Here, the underlying mechanism explaining the differential impact seems rather straightforward: lower socioeconomic groups often have less income and a smaller budget to spend on foods than higher socioeconomic groups. Therefore, lower socioeconomic groups are likely more susceptible to policies that increase the prices of unhealthy foods, or financial support that increases their budget to spend on healthy foods. However, for other food environmental policies, evidence is scarce and potential underlying mechanisms are less clear.

Lower and higher socioeconomic groups not only differ in the healthfulness of their dietary intakes, but also in the material and sociocultural circumstances in which they are born, grow up, work and age, i.e. their daily living conditions (e.g. income, housing, wealth).^12^ We argue that an application of theories explaining socioeconomic inequalities in health—which specify the role of specific elements in these daily living conditions—may help to increase our understanding via which underlying mechanisms food environment policies differentially affect lower and higher socioeconomic groups. Applying these theories is likely to provide better insights into the ultimate causes of socioeconomic inequalities in diets, and how these may affect the impact of food environment policies. While the recently published Nutrition Equity Framework also explicitly incorporates the idea of capitals and intergenerational equity shaping daily living conditions and influencing dietary intakes,^12^ most theories for explaining healthy dietary intakes have mainly focused on the more proximal determinants of food choices, such as knowledge, attitudes and self-efficacy towards healthy food consumption (as specified in health behaviour theories like the Theory of Planned Behaviour).

To illustrate our point, we considered two theories that can be applied to explain socioeconomic inequalities in health which have gained momentum over recent years and are particularly helpful to understand inequalities in ‘health-behaviours’ (including broader lifestyle behaviours):^13–20^ Bourdieu’s capital theory and Mullainathan and Shafir’s scarcity theory. We first shortly introduce the capital theory, and then apply this theory by means of illustration, to one Food-EPI domain, namely food promotion policies. We then introduce the scarcity theory and apply it by means of illustration, to another Food-EPI domain, namely food labelling policies. We conclude with implications for research and practice.

### Bourdieu’s capital theory and the concept of habitus and distinction

According to Bourdieu, capital is accumulated labour (in materialized or embodied forms) that enables individuals to maintain and enhance their position in the social world.^13^^,^^21^ Bourdieu distinguishes three forms of capital: economic, social and cultural capital.^13^^,^^21^ Economic capital refers to material resources, i.e. money and other assets such as property rights.^13^ Social capital refers to the idea that social networks are a potential resource for individuals, communities and society.^18^ Cultural capital refers to the operational skills, linguistic styles, values and norms that one acquires through education and lifelong socialization.^22^ Cultural capital comes in three forms: incorporated cultural capital (e.g. norms, values, knowledge), objectivized cultural capital (e.g. books, tools) and institutionalized cultural capital (e.g. educational degrees).^22^ Incorporated cultural capital, e.g. ‘long-lasting dispositions of the mind and the body’, includes (health) values, norms, perceptions, skills, and knowledge acquired through a lifelong socialization process.^21^^,^^23^ Via socialization, these norms, values, preferences and habits become internalized as part of a broader ‘habitus’,^21^ which is another important concept of Bourdieu’s capital theory and plays an important role in the establishment of lifestyles.^15^^,^^23^ This ‘habitus’ can be understood as an embodied arrangement of social structures that predisposes an individual to certain actions^24^ in accordance with the social context in which it is produced.^19^ Habitus expresses itself in all domains of life: in aesthetic preferences, cultural practices, as well as choices related to health behaviour and lifestyles.^14^ According to Bourdieu, members of the same social groups often share a similar position in social space with an affinity in lifestyles between them, which may become part of an identity and is used as a ‘distinction mechanism’, reflecting differences between social groups.^21^ Recent studies have provided evidence that higher socioeconomic groups may indeed be more likely to adopt a healthy lifestyle as an expression of ‘social distinction’, which includes a healthy consumption pattern (e.g. eating recommended levels of fruits and vegetables everyday).^16^^,^^25^ Importantly however, the impact of the habitus on broader lifestyles, which may for instance lead to socioeconomic differences in types of media used,^26^ may also be an important mechanism through which socioeconomic groups are differentially exposed to (online) food environments (e.g. advertising for fast food).

### Bourdieu and policies restricting unhealthy food marketing

‘Food promotion’ is one of the policy domains in the Food-EPI framework and concerns policies that restrict or ban the promotion of unhealthy foods to children and adolescents through broadcast media (television, radio), social and online media, and non-broadcast media (e.g. sport and cultural events, magazines).^7^ Such policies are important for the healthy dietary intakes of children and adolescents, as studies have shown that marketing of unhealthy foods encourages purchase requests of children and adolescents towards unhealthy foods,^27^ leading to a higher consumption of unhealthy foods (e.g. sweet and salty snacks, fast foods) and a lower consumption of healthy foods (e.g. fruit and vegetables).^27^

A digital divide, in which those with more economic capital had more access to new forms of media, has now been replaced by a media-dominated society to which both higher and lower socioeconomic groups are exposed. Food environments are also rapidly digitalizing and digital food marketing has become widespread using a range of techniques (e.g. advergaming, harvesting personal data from digital platforms, online brand consumer engagement).^28^ From the perspective of Bourdieu, television and internet use for leisure purposes ‘trickled down’ as cultural goods, and now contribute to the formation of cultural capital. Indeed, ‘not watching broadcast television’ or ‘watching specific programmes or channels’ might be seen as a way to create distinction. Similarly, people can distinguish themselves via the use of non-broadcast media such as reading specific magazines or attending certain events (e.g. sports, cultural events). Media exposure increasingly can be seen a ‘classifying practice’,^29^ in which persons occupying different positions in the space of social positions, select and use media differently.

Thus, differences in cultural capital between higher and lower socioeconomic groups may lead to different food and media preferences. This information is used by the industry for tailor-made food marketing strategies (including when, where and which foods are advertised),^30^ leading to a higher exposure of lower socioeconomic groups to unhealthy food marketing.^31^ Moreover, exposure to (digital) food marketing may subsequently contribute to the habitus by influencing food choices, preferences and consumption^27^ leading to a reinforcing feedback mechanism. This illustrates that different elements of the living conditions (e.g. social practices, habitus, media use, exposure to food marketing, food consumption) are interconnected, and that changes in one element affect other parts of the system via operating feedback loops, resulting in certain dietary behaviours of lower and higher socioeconomic groups.^32^

Thus, food promotion policies that restrict or ban the promotion of unhealthy foods may protect children and adolescents across all population groups. Moreover, these policies can limit the potential of marketing to be a classifying practice and contribute to breaking the vicious circle described above. This is because, as a result of differences in elements of their living conditions (e.g. social practices, habitus, media use) lower socioeconomic groups may have a higher exposure to unhealthy food marketing than higher socioeconomic groups which in turn influences food preferences. Therefore, policies banning the promotion of all unhealthy foods or targeting foods or media for which especially lower socioeconomic groups have a preference, may especially protect these groups and eventually lead to a reduction of socioeconomic inequalities in dietary intakes.

### The scarcity theory

According to the scarcity theory, the scarcity mindset entails a feeling of not having enough of something, e.g. money or time.^33^ The feeling of scarcity comes from having limited resources in terms of money or time, but also from the subjective perception of what matters (e.g. how important a certain purchase is, or which tasks really need to be accomplished within a certain time frame). Scarcity can capture the mind and change how people think,^33^ it may lead to less ‘cognitive bandwith’ resulting in a neglect of other concerns that may feel less urgent. Unfavourable daily living conditions (e.g. financial debts, deprived housing conditions, social problems) are more prevalent in lower socioeconomic groups, leading to a higher prevalence of scarcity in lower as compared to higher socioeconomic groups.^17^ The stress resulting from the experience of scarcity can lead to losing the capacity to give long term goals, such as optimal health, their full consideration, as the mind is fully occupied with more urgent concerns.^34^ Empirical evidence shows that experiencing scarcity for a longer period of time (at least two years) increases the consumption of discretionary calories (including those from industrially processed foods high in sodium, added sugar or saturated fat) and reduces the consumption of fruit and vegetables.^17^

### Scarcity and food labelling policies

The Food-EPI domain of food labelling concerns policies that require food producers to put nutrient information, ingredient lists or front-of-pack labels (like the traffic-light system) on packaged foods. Such information is thought to help consumers to be better able to make informed, healthy food choices, and therefore may promote healthier dietary intakes.^35^ However, such information has found to be less used by people with a lower than people with a higher socioeconomic position^36^ (although evidence is inconclusive^37^). The scarcity theory can provide insights into potential underlying mechanisms for these socioeconomic inequalities in the use of nutrient information and front-of-pack labels.

Since the lists and declarations on food products are often not easy to read or understand, one need to deliberately dedicate time and cognitive energy to read the labels, process its information, compare it to the nutrient information on alternative food products, and finally make an informed choice on which products to buy. Individual agency thus plays a large role for these policies to have a positive effect on the healthfulness of diets.^8^ This agency for making such informed choices may be constrained by scarcity especially experienced by members of lower socioeconomic groups, as their minds are occupied with urgent concerns related to their less favourable daily living conditions (e.g. financial debts, deprived housing conditions). Less cognitive bandwith is then available for pondering over healthy and unhealthy food choices, and for dedicating precious time and energy for processing nutrient information to be used for deliberate healthy choices.

Clearly, front-of-pack labels may be easier to read and understand than classical lists and declarations,^38^ but they still require individual agency to deliberately read these labels and choose to buy healthier foods and not buy unhealthy foods. For instance, parents with a lower socioeconomic position may deliberately choose to buy unhealthy foods to compensate for other domains of scarcity, thereby satisfying their children’s requests for the unhealthy foods they like, and bolstering their sense of worth as caregivers.^25^ In addition, people experiencing scarcity may not want to waste time and resources buying and preparing healthy foods that their children will not eat, and thus choose for unhealthy foods satisfying children’s likes and dislikes.^39^

Thus, it is likely that socioeconomic differences in daily living conditions that lead to higher levels of experienced scarcity in lower socioeconomic groups may result in food labelling policies having more beneficial effects on the diets of higher than lower socioeconomic groups, and therefore may lead to a widening of dietary inequalities.

## Conclusions

In this paper, we showed how two theories that have been increasingly used over the past years for explaining socioeconomic inequalities in health also can help to understand how food environment policies may impact lower and higher socioeconomic groups differently. Some of these food environment policies are so called ‘agento-structural’ policies. These require at least a certain amount of individual agency to result in a positive effect on the healthfulness of diets,^8^ and therefore could unintentionally increase socioeconomic inequalities in healthy food consumption. We reasoned that, due to generally less favourable daily living conditions of lower socioeconomic groups (e.g. higher levels of financial scarcity), individual agency to make healthy food choices is constrained. As a result, diets of lower socioeconomic groups less likely benefit from ‘agento-structural’ food environmental policies (like food labelling) than higher socioeconomic groups, and thus lead to a widening of inequalities. Other, more ‘structural’ type of food environment policies require little agency of individuals and lead to an improvement of the unfavourable food environmental features to which lower socioeconomic groups are more often exposed.^8^ These structural policies are more likely to decrease socioeconomic inequalities in healthy food consumption. For instance, we reasoned that, due to specific elements in daily living conditions (e.g. social practices, habitus, media use), lower socioeconomic groups may have a higher exposure to unhealthy food marketing which in turn influences food preferences. As a result, policies restricting the promotion of unhealthy food products and with that limiting the potential of marketing to be a classifying practice, can be especially beneficial for lower socioeconomic groups.

Some limitations of our approach and reasoning should be acknowledged. In our illustration, we applied one theory to one food environment policy domain, and described potential mechanisms according to that specific theory. Certainly, this is an oversimplification of reality as food environment policies do not happen in a vacuum. In real life, also other factors than these theory-specific factors are at play, and multiple (food environment) policies may interact with each other in their effect on diets and health. In order to take into account the complexities of real life in the best possible way, the application of a systems perspective to account for the most important factors involved in the explanation of inequalities in dietary intake—as well as how these factors interact—is extremely important.^32^ Further, applying different theories for explaining health inequalities, may emphasize other underlying mechanisms on how food environment policies may contribute to a reduction or increase in socioeconomic inequalities in diet. For instance, the scarcity theory may emphasize more the less favourable daily living conditions of lower socioeconomic groups (e.g. higher levels of financial scarcity), while Bourdieu’s capital theory emphasizes specific elements in daily living conditions of both lower as well as higher socioeconomic groups (e.g. social practices, habitus). The results of different studies, applying different health inequality-theories, testing different hypotheses will only strengthen the evidence base and lead to richer insights in the most promising food environment policies for reducing dietary inequalities. The application of theories that explicitly take into account the daily living conditions of different socioeconomic groups can have important implications for future research and practice:

- Theories used for explaining health inequalities can help to formulate innovative, theory-based hypotheses on the differential impact of food environment policies that could be tested in future research (e.g. studies on how front-of-pack labelling differentially impacts lower and higher socioeconomic groups with different exposures to scarcity).
- Hypotheses on the impact of food environment policies on diets, based on theories that take elements of broader daily living conditions into account (e.g. the social practices that lead to habitus formation, the material conditions that shape experiences of scarcity) may potentially be more capable of forecasting their potential modest impacts.
- To reduce dietary inequalities, food environment policies tackling proximal determinants of dietary behaviour (e.g. knowledge on food ingredients) should be aligned and combined with other policies, tackling more distal determinants of unhealthy diets (e.g. financial debts, deprived housing conditions, media exposure, social problems). As only then their effects are reinforced, and only then considerable decreases in inequalities may be expected.
- To account for all the determinants involved in the numerous underlying mechanisms between socioeconomic position and dietary intakes—including food environment exposures, living conditions and individual-level factors—the application of a systems perspective (a system of multiple, interconnected factors exerting non-linear influence on dietary intakes), can enhance the development of effective policies tackling socioeconomic inequalities in dietary intakes.^32^
